# Derivation and Validation of a Score for Predicting Poor Neurocognitive Outcomes in Acute Carbon Monoxide Poisoning

**DOI:** 10.1001/jamanetworkopen.2022.10552

**Published:** 2022-05-05

**Authors:** Sung Hwa Kim, Yoonsuk Lee, Soo Kang, Jin Hui Paik, Hyun Kim, Yong Sung Cha

**Affiliations:** 1 Department of Biostatistics and Center of Biomedical Data Science, Yonsei University Wonju College of Medicine, Wonju, Republic of Korea; 2 Department of Emergency Medicine, Yonsei University Wonju College of Medicine, Wonju, Republic of Korea; 3 Research Institute of Hyperbaric Medicine and Science, Yonsei University Wonju College of Medicine, Wonju, Republic of Korea; 4 Department of Emergency Medicine, Inha University College of Medicine, Incheon, Republic of Korea

## Abstract

**Importance:**

Preventing neurocognitive sequelae is a major goal of treating acute carbon monoxide (co) poisoning. There is a lack of reliable score systems exist for assessing the probability of these sequelae.

**Objective:**

To develop and validate a novel clinical scoring system for predicting poor neurocognitive outcomes after acute co poisoning.

**Design, Setting, and Participants:**

This prognostic study included derivation and validation cohorts based on consecutive patient data prospectively collected at university hospitals from January 2006 to July 2021 in Wonju, Republic of Korea, and from August 2016 to June 2020 in Incheon, Republic of Korea. Participants included individuals aged 16 years or older admitted with co poisoning. Data were analyzed from October 2021 to January 2022.

**Exposures:**

Clinical and laboratory variables.

**Main Outcomes and Measures:**

The outcome of interest was neurocognitive sequelae at 4 weeks after co poisoning. Logistic regression models were used to identify predictors of poor neurocognitive outcomes in the derivation cohort. Outcomes were assessed using the Global Deterioration Scale [GDS] at 1-month after co exposure and classified as good (1-3 points) or poor (4-7 points).

**Results:**

A total of 1282 patients (median [IQR] age, 47.0 [35.0-59.0] years; 810 [63.2%] men) were assessed, including 1016 patients in the derivation cohort and 266 patients in the validation cohort. The derivation cohort included 126 patients (12.4%) with poor GDS scores. Among 879 patients in the derivation cohort with 1-year follow-up data, 757 (86.1%) had unchanged GDS scores, 102 (11.6%) had improved GDS scores, and 20 (2.3%) had worsened GDS scores. In the final prediction model, age older than 50 years (1 point), Glasgow Coma Scale score of 12 or less (1 point), shock (1 point), serum creatine kinase level greater than 320 U/L at emergency department presentation (1 point), and no use of hyperbaric oxygen therapy (1 point) remained factors significantly associated with worse outcome; therefore, this scoring system was called *COGAS* (creatine kinase, hyperbaric oxygen therapy, Glasgow Coma Scale, age, shock). Area under the receiver operating characteristic curve for COGAS score was 0.862 (95% CI, 0.828-0.895) for the derivation cohort and 0.870 (95% CI, 0.779-0.961) for the validation cohort.

**Conclusions and Relevance:**

These findings suggest that assessing the COGAS score during the early phase of co poisoning may help identify patients at risk of poor neurocognitive sequelae.

## Key Points

**Question:**

Can a novel clinical scoring system predict poor neurocognitive outcomes after acute carbon monoxide poisoning?

**Findings:**

This prognostic study developed and externally validated a prediction model including 5 risk factors associated with poor neurocognitive outcome at 1 month, creatine kinase level, hyperbaric oxygen therapy, Glasgow Coma Scale score, age, and shock (COGAS score), among patients with carbon monoxide poisoning. COGAS score showed excellent discrimination performance.

**Meaning:**

These findings suggest that use of a reliable prediction model during the early phase of carbon monoxide poisoning could help identify patients at risk of poor neurocognitive sequelae.

## Teaser

This prognostic study develops and validates a clinical scoring system for predicting poor neurocognitive outcomes after acute carbon monoxide poisoning.

## Introduction

Annually, approximately 50 000 patients with carbon monoxide (co) poisoning present to emergency departments (EDs) of US hospitals, with 1500 deaths.^1,2,3^ Approximately 15 000 intentional co poisonings annually account for more than two-thirds of reported deaths^2,4,5^ and cause neurocognitive sequelae among survivors.^6,7^ Of patients with co poisoning treated with normobaric oxygen, nearly half develop cognitive sequelae after 6 weeks.^6^ Some possibly permanent sequelae include gait and motor disturbances, peripheral neuropathy, hearing loss and vestibular abnormalities, dementia, and psychosis.^7^ The societal costs (in direct hospital costs and lost earnings) of accidental co poisoning are estimated at more than $1.3 billion annually in the US.^8^ Hyperbaric oxygen (HBO) therapy within 24 hours after poisoning is recommended for symptomatic patients with co poisoning.^1,6,9,10^ In a 2002 study by Weaver,^6^ treatment with HBO reduced neurocognitive sequelae incidence at 6 weeks by half and from 25% to 18% at the 1-year evaluation, compared with no such treatment. However, neurocognitive sequelae developed in 18% of patients even with HBO use in acute co poisoning. It is important to be able to predict a poor neurocognitive prognosis in acute co poisoning to initiate rehabilitative interventions early.^11^

Clinical and laboratory variables suggested for predicting poor neurocognitive prognosis^6,7,12,13,14,15,16,17,18,19,20^ include older age, underlying cardiovascular disease, any interval loss of consciousness, acidosis, Glasgow Coma Scale (GCS) score, serum creatine kinase level, longer co exposure intervals, serum lactate level, and HBO treatment. As there is no validated simple and accurate clinical scoring model for stratifying patients according to their risk of poor neurocognitive prognosis in early phase, we aimed to develop and validate a simple, accurate clinical scoring model for predicting poor neurocognitive outcomes at 1 month after co poisoning.

## Methods

This prognostic study was approved by the institutional review boards at Wonju Severance Christian Hospital and Inha University Hospital. In the derivation cohort, data after August 2020 were collected with informed consent for a prospective cohort study. For data before August 2020 in the derivation cohort and all data in the validation cohort, the requirement for informed consent was waived because we analyzed retrospectively with prospectively collected registry data. This study adhered to the ethical guidelines of the Declaration of Helsinki.^21^ This study followed the Transparent Reporting of a Multivariable Prediction Model for Individual Prognosis or Diagnosis (TRIPOD) reporting guideline.

### Study Cohorts

The study data were derived from independent cohorts at 2 tertiary academic hospitals in the Republic of Korea. The derivation cohort included those with acute co poisoning enrolled between January 2006 and July 2021 at Wonju Severance Christian Hospital, where a co poisoning registry was opened to prospectively collect consecutive patient data. Data from January 2006 to July 2020 were prospectively obtained from this registry; after August 2020, data were collected prospectively after individuals or legal guardians provided their informed consent for the Carbon Monoxide Intoxication in Korea: Prospective Cohort (CARE CO Cohort; ClinicalTrials.gov identifier: NCT04490317). Patients were followed up until August 2021. The validation cohort included registry data from patients with co poisoning collected prospectively from August 2016 to June 2020 at the Inha University Hospital, Incheon. These institutions have previously interacted to conduct various multicenter studies on co poisoning; therefore, the patients were registered using similar registration forms. Inha University Hospital has been implementing this since 2016. We anonymized patients’ data before analyses.

At both hospitals, acute co poisoning is diagnosed based on patient history and carboxyhemoglobin (Hbco) level of greater than 5% (>10% for smokers; to convert to proportion of 1.0, multiply by 0.01). We treated patients with co poisoning with 100% oxygen therapy through a face mask with a reservoir bag. Patients with any loss of consciousness interval, neurocognitive symptoms and signs, cardiovascular dysfunction, elevated cardiac enzymes, ischemic electrocardiogram changes, severe acidosis, or Hbco of 25% or greater were treated with HBO.^1^ During the first HBO session, initial compression was performed to 2.8 atmospheres absolute for 45 minutes, followed by 2.0 atmospheres absolute for 60 minute, similar to the protocol described by Thom et al.^10^

### Data Extraction

For widespread applicability, we limited variables to known and potential risk factors^6,7,12,13,14,15,16,17,18,19,20^ and commonly used variables in acute co poisoning. We evaluated patient age, sex, poisoning intentions, co source (ie, charcoal, oil and gas, or fire), drug coingestion, GCS score at the site of rescue or ED arrival, comorbidities (ie, diabetes, hypertension, cardiovascular disorder, and psychiatric disease), current smoking status and alcohol coingestion, interval of loss of consciousness, shock, seizure, and application of HBO (eAppendix 1 in the Supplement). We excluded co exposure time from the main scoring model, requiring only objective indicators, as it is difficult to measure co exposure time accurately in clinical settings for many patients. Laboratory variables assessed in the ED included Hbco, bicarbonate, lactate, creatinine, creatine kinase, and troponin I levels. We excluded patients with missing clinical and laboratory values.

co-related neurocognitive outcomes were measured using the Global Deterioration Scale (GDS) score (range, 1-7) (eAppendix 2 and eTable in the Supplement).^22^ For patients with GDS scores of 1 to 3 points, cognitive impairment ranged from none (GDS score, 1 point) to mild (GDS score, 3 points), objectively substantiated by a detailed interview by an experienced rehabilitation physician. Patients with GDS scores of 4 to 7 points ranged from clear cognitive impairment (GDS score, 4 points) to loss of motor skills (including walking) and loss of all language skills except for inaudible, unintelligible sounds (GDS score, 7 points). Patients who died within 1 month were assigned a GDS score of 7 points, the most severe score. We investigated GDS stage at 1 month after co exposure through visiting the rehabilitation outpatient department. We assumed neurocognitive sequelae caused by co poisoning rarely develop after 4 to 6 weeks.^6,7,10,23,24^ For patients unable to visit outpatient departments, their guardians were interviewed. We classified GDS scores as good (1-3 points) or poor (4-7 points)^25^ and investigated changes in the 1-month GDS score at 1 year after co poisoning.

### Variable Selection and Score Construction

For the practical application of scores, a receiver operating characteristic curve analysis was performed to determine the cutoff value for converting a continuous variable to a categorical variable (eFigure 1 in the Supplement). Using MedCalc version 11.6.1 (MedCalc Software), the cutoff values were obtained using the maximum sensitivity and specificity based on the Youden Index. To minimize potential collinearity and overfitting of variables, multivariable logistic regression using backward elimination included variables at *P* < .05 in the univariate analysis.^26^ Finally, 5 variables were included in the scoring system, the accuracy of which was assessed by the area under the receiver operating characteristic curve (AUC). Model calibration was assessed using the Hosmer-Lemeshow goodness of fit test, and calibration plots were visually evaluated using 200 bootstrap resamples.

### Internal Validation

To reduce overfit bias, internal validation of the prediction model was assessed using 2 methods: 10-fold cross-validation and bootstrapping method (200 iterations). The 10-fold cross-validation was used by dividing the training set into 10 mutually exclusive parts. Consequently, the predictive performance of COGAS score was reassessed with 200 iterations of bootstrap resampling. The bootstrapping method can be constructed and validated by 100% of all patients and is more efficient than validation methods using split.^27^

### External Validation

After developing the scoring system, external validation was performed on different data. The observed and estimated probabilities of poor neurocognitive outcomes were compared in each COGAS score. Then, the discrimination was assessed using the AUC, and calibration was assessed using the calibration plot.

### Statistical Analysis

Data are reported as means with SDs for continuous variables and numbers with percentages for categorical variables. Differences between the derivation and validation cohorts were assessed using the independent *t* test or Mann-Whitney *U* test for continuous variables and χ^2^ test or Fisher exact test for categorical variables. DeLong test was used to confirm whether the AUCs of the 2 models were statistically significantly different. All statistical analyses used SAS statistical software version 9.4 (SAS Institute) and R version 3.6.3 (R Project for Statistical Computing). A 2-sided *P* < .05 indicated statistical significance. Data were analyzed from October 2021 to January 2022.

## Results

### Study Population

A total of 1282 patients (median [IQR] age, 47.0 [35.0-59.0] years; 810 [63.2%] men) were assessed, including 1016 patients in the derivation cohort and 266 patients in the validation cohort (eFigure 2 in the Supplement). The derivation cohort included 126 patients (12.4%) with poor GDS scores (Table 1). The median (IQR) age in the derivation cohort was 48.0 (36.0-60.5) years, and 635 (62.5%) were men. Charcoal (758 patients [74.6%]) was the most common co source, and the median (IQR) GCS score at the scene or ED was 15.0 (12.0-15.0). Hypertension (197 patients [19.4%]) was the most common comorbidity, and HBO therapy was provided to 869 patients (85.5%). Compared with the validation cohort, patients in the derivation cohort were younger, were less likely to receive HBO therapy, and had lower creatine kinase levels, whereas they were more likely to have intentional co poisoning, drug coingestion, psychiatric disorders, higher lactate levels, and higher troponin I levels (Table 1). Overall, 15 patients (1.2%) died within 1 month after co poisoning while hospitalized. Cause of death was uncontrolled metabolic acidosis, acute kidney injury, or profound shock, except for 1 patient with aortic dissection and who was bedridden (GDS score, 7 points). We investigated neurological impairment because GDS may be difficult to reflect it. In our study, among 9 patients with neurological symptoms (motor weakness, speech disturbance, and peripheral neuropathy, such as foot drop), 3 had poor outcomes (with GDS scores of 4-6), 5 had good outcomes (initial neurological symptoms resolved), and the remaining patient in the favorable outcome group had initial persistent foot drop (peripheral neuropathy) at 1 month, which returned to normal at 10 months after co poisoning.

**Table 1.** Baseline Characteristics of Patients in the Derivation and Validation Cohorts

Variables	Patients, No. (%)			*P* value
	Total (N = 1282)	Derivation cohort (n = 1016)	Validation cohort (n = 266)	
Age, median (IQR), y	47.0 (35.0-59.0)	48.0 (36.0-60.5)	44.0 (31.0-55.0)	<.001
Sex				
Women	472 (36.8)	381 (37.5)	91 (34.2)	
Men	810 (63.2)	635 (62.5)	175 (65.8)	.32
Intentionality	520 (40.6)	381 (37.5)	139 (52.3)	<.001
co source				
Charcoal	952 (74.3)	758 (74.6)	194 (72.9)	.40
Gas	167 (13.0)	126 (12.4)	41 (15.4)	
Fire	163 (12.7)	132 (13.0)	31 (11.7)	
Drug coingestion	124 (9.7)	73 (7.2)	51 (19.2)	<.001
GCS score, median (IQR)	15.0 (12.0-15.0)	15.0 (12.0-15.0)	15 (13.0-15.0)	.006
Comorbidities				
Diabetes	136 (10.6)	111 (10.9)	25 (9.4)	.47
Hypertension	238 (18.6)	197 (19.4)	41 (15.4)	.14
Cardiovascular disease	50 (3.9)	43 (4.2)	7 (2.6)	.23
Psychiatric disease	180 (14.0)	126 (12.4)	54 (20.3)	.001
Alcohol coingestion	225 (17.6)	182 (17.9)	43 (15.8)	.51
Current smoker	503 (39.2)	387 (38.1)	116 (43.6)	.10
Symptoms at ED				
Loss of consciousness	767 (59.8)	618 (60.8)	149 (56.0)	.15
Shock	50 (3.9)	36 (3.5)	14 (5.3)	.20
Seizure	17 (1.3)	13 (1.3)	4 (1.5)	.77
Use of HBO	1070 (83.5)	869 (85.5)	201 (75.6)	<.001
Laboratory findings, median (IQR)				
Hbco, %	19.0 (7.5-31.4)	18.7 (7.4-30.4)	21.3 (8.3-35.2)	.07
Bicarbonate, mEq/L	21.4 (18.8-23.4)	21.4 (18.8-23.3)	22.0 (18.0-24.0)	.17
Lactate, mg/dL	20.72 (12.61-32.43)	18.92 (12.61-31.53)	23.42 (14.41-44.14)	<.001
Creatinine, mg/dL	0.8 (0.7-1.0)	0.8 (0.7-1.0)	0.8 (0.7-1.1)	.09
Creatine kinase, U/L	146.5 (92.0-360.0)	149.0 (94.5-393.0)	131.5 (87.0-298.0)	.02
Troponin I, ng/mL	0.07 (0.02-0.32)	0.02 (0.02-0.33)	0.1 (0.10-0.26)	<.001
GDS				
Good (1-3)	1129 (88.1)	890 (87.60)	239 (89.9)	.31
Poor (4-7)	153 (11.9)	126 (12.4)	27 (10.2)	

Abbreviations: co, carbon monoxide; Hbco, carboxyhemoglobin; ED, emergency department; GCS, Glasgow Coma Scale; GDS, Global Deterioration Scale; HBO, hyperbaric oxygen.

SI conversion factors: To convert bicarbonate to millimoles per liter, multiply by 1; creatine kinase to microkatals per liter, multiply by 0.0167; creatinine to micromoles per liter, multiply by 76.25; lactate to millimoles per liter, multiply by 0.111; Hbco to proportion of 1.0, multiply by 0.01; troponin I to micrograms per liter, multiply by 1.

We investigated whether the 1-month GDS score changed after 1 year (879 patients were followed-up for 1 year) after co poisoning in the derivation cohort. In 757 patients (86.1%) GDS scores remained unchanged, 102 patients (11.6%) had improved GDS scores, and 20 patients (2.3%) had worse GDS scores.

### Factors Associated With Poor Neurocognitive Outcomes

In univariate analysis, all variables except sex, drug coingestion, cardiovascular disease, psychiatric disorder, alcohol coingestion, and seizure were associated with poor neurocognitive outcomes. In multivariable analysis, intentionality, co source, diabetes, hypertension, current smoker status, loss of consciousness, Hbco, and levels of serum bicarbonate, lactate, creatinine, and troponin I were not statistically significant.

Model 1 (main model) included creatine kinase (laboratory value). Model 2 excluded the laboratory variable of model 1, which may not be immediately available and allows a system without laboratory measurements.

In model 1, age older than 50 years (odds ratio [OR], 4.06; 95% CI, 2.53-6.53), GCS score of 12 or less (OR, 6.06; 95% CI, 3.44-10.68), shock (OR, 3.65; 95% CI, 1.64-10.68), no HBO treatment (OR, 2.41; 95% CI, 1.32-4.40), and serum creatine kinase at the ED greater than 320 U/L (to convert to microkatals per liter, multiply by 0.0167; OR, 5.06; 95% CI, 3.15-8.13) remained significant (Table 2). We named this scoring system COGAS for the 5 elements: creatine kinase, hyperbaric oxygen therapy, Glasgow Coma Scale score, age, and shock. The distribution of COGAS score in the cohorts is presented in eFigure 3 in the Supplement). In multivariable analysis in model 2, poor neurocognitive outcomes occurred in patients aged older than 50 years (OR, 4.27; 95% CI, 2.71-6.74), with GCS scores of 12 or less (OR, 10.49; 95% CI, 6.12-17.99), with shock (OR, 4.55; 95% CI, 2.09-9.90), and without HBO treatment (OR, 2.49; 95% CI, 1.42-4.36).

**Table 2.** Factors Associated With Poor Neurocognitive Outcomes in the Derivation Cohort

Variables	OR (95% CI)		Score
	Unadjusted (model 1)	Adjusted (model 2)	
Age >50 y	3.53 (2.35-5.31)	4.06 (2.53-6.53)	1
Male sex	0.87 (0.59-1.27)	NA	NA
Intentionality	1.50 (1.03-2.18)	NA	NA
Source			
Charcoal	1 [Reference]		NA
Oil and gas	0.29 (0.12-0.67)	NA	NA
Fire	0.37 (0.18-0.78)	NA	NA
Drug coingestion	0.99 (0.48-2.05)	NA	NA
GCS score ≤12	11.12 (6.62-18.65)	6.06 (3.44-10.68)	1
Comorbidities			
Diabetes	1.78 (1.06-2.98)	NA	NA
Hypertension	2.17 (1.44-3.28)	NA	NA
Cardiovascular disease	1.66 (0.75-3.66)	NA	NA
Psychiatric disease	1.40 (0.83-2.35)	NA	NA
Alcohol coingestion	1.03 (0.48-2.22)	NA	NA
Current smoker	0.59 (0.39-0.89)	NA	NA
Symptoms at ED			
Loss of consciousness	6.63 (3.68-11.94)	NA	NA
Shock	11.67 (5.84-23.32)	3.65 (1.64-10.68)	1
Seizure	3.21 (0.97-10.58)	NA	NA
No use of HBO	2.07 (1.31-3.25)	2.41 (1.32-4.40)	1
Laboratory findings			
Hbco >37.4%	1.80 (1.13-2.88)	NA	NA
Bicarbonate ≤19.5 mEq/L	3.58 (2.44-5.24)	NA	NA
Lactate >18.02 mg/dL	2.35 (1.56-3.53)	NA	NA
Creatinine >1.0 mg/dL	4.46 (3.03-6.56)	NA	NA
Creatine kinase >320 U/L	9.84 (6.43-15.04)	5.06 (3.15-8.13)	1
Troponin I >0.113 ng/mL	8.66 (5.58-13.44)	NA	NA

Abbreviations: Hbco, carboxyhemoglobin; ED, emergency department; GCS, Glasgow Coma Scale; HBO, hyperbaric oxygen; NA, not applicable; OR, odds ratio.

SI conversion factors: To convert bicarbonate to millimoles per liter, multiply by 1; creatine kinase to microkatals per liter, multiply by 0.0167; creatinine to micromoles per liter, multiply by 76.25; lactate to millimoles per liter, multiply by 0.111; Hbco to proportion of 1.0, multiply by 0.01; troponin I to micrograms per liter, multiply by 1.

### Performance and Calibration of the COGAS Score

**Figure 1.**
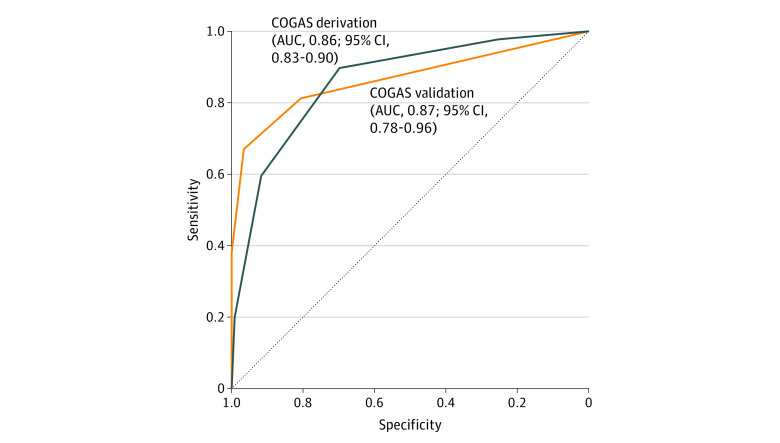
Area Under the Receiver Operating Characteristic Curve (AUC) of the Score Model in the Derivation and Validation Cohorts COGAS indicates the prediction score calculated on creatine kinase, hyperbaric oxygen therapy, Glasgow Coma Scale, age, and shock.

The AUC for COGAS score was calculated for both cohorts (Figure 1). For the derivation cohort, the AUC was 0.862 (95% CI, 0.828-0.895), and for the validation cohort, the AUC was 0.870 (95% CI, 0.779-0.961), indicating excellent discriminatory performance (Figure 1). The Hosmer-Lemeshow goodness of fit test yielded χ^2^ = 2.61 (*P* = .27). Internal validation of COGAS score demonstrated good discrimination in the derivation cohort databased 10-fold cross-validation analysis (AUC, 0.864; 95% CI, 0.852-0.875) and the bootstrapping method (AUC, 0.862; 95% CI, 0.829-0.896). Calibration plots of COGAS score for each cohort showed good agreement between the predicted and observed outcomes, with close approximation (eFigure 4 in the Supplement). The original intercept and slope of the calibration plots were 0 and 1, respectively.

We compared the models, and model 2 score still revealed a high discrimination (AUC, 0.812; 95% CI, 0.787-0.836) compared with model 1 (0.862). The performance of the models developed with and without creatine kinase (a laboratory value) was significantly different (eFigure 5 in the Supplement).

### Scoring Model Validation

**Figure 2.**
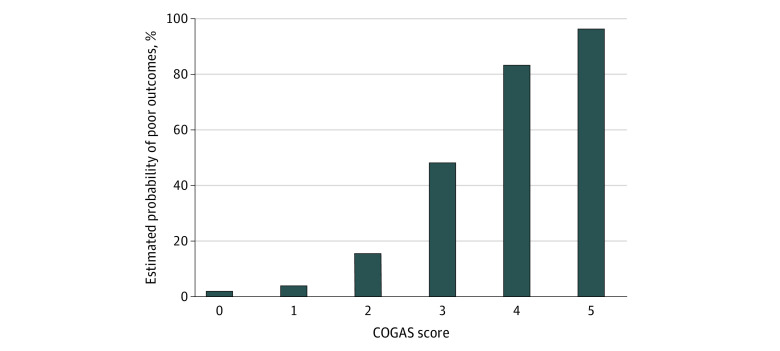
Estimated Probability of 1-Month Poor Neurocognitive Outcomes in Patients With Acute Carbon Monoxide Poisoning COGAS indicates the prediction score calculated on creatine kinase, hyperbaric oxygen therapy, Glasgow Coma Scale, age, and shock.

The observed 1-month probabilities of poor neurocognitive outcomes based on COGAS score were similar to those of the derivation and validation cohorts (Table 3; eFigure 6 in the Supplement). The estimated 1-month probabilities of COGAS score–based poor neurocognitive outcomes were: 0% for a score of 0, 3.2% for a score of 1, 15.1% for a score of 2, 48.4% for a score of 3, 83.2% for a score of 4, and 96.3% for a score of 5 (Figure 2). The observed and estimated probabilities of poor neurocognitive outcome were strongly correlated (*r* = 0.989; *R*^2^ = 0.978; *P* < .001). Similarly, model 2 was well calibrated and validated and, thus could be used without laboratory variables (eFigure 7 and eFigure 8 in the Supplement).

**Table 3.** Poor Outcomes Evidenced by COGAS Score in the Derivation and Validation Cohorts^a^

COGAS score	Derivation cohort (n = 1016)		Validation cohort (n = 266)	
	Poor outcomes, No./total No.	Observed poor outcomes, %	Poor outcomes, No./total No.	Observed poor outcomes, %
0	3/237	1.3	2/86	2.3
1	10/398	2.5	3/112	2.7
2	38/233	16.3	4/43	9.3
3	50/116	43.1	8/14	57.1
4	20/26	76.9	7/8	87.5
5	5/6	83.3	3/3	100.0

Abbreviation: COGAS, creatine kinase, hyperbaric oxygen therapy, Glasgow Coma Scale, age, shock.

^a^ The probability of poor outcomes 1 month after carbon monoxide exposure increased significantly with increasing COGAS scores (*P* for trend < .001).

### Additional Analysis

We compared the diagnostic power of the model including co exposure time compared with model 1 in the derivation cohort. The explanatory power of model 1 (0.862) was similar to that of the model with co exposure time (AUC, 0.869; 95% CI, 0.836-0.902; *P* = .31) (eFigure 9 in the Supplement). In addition, we analyzed the verification power of COGAS score in patients with poisoning by oil and gas combustion, which are the main sources of co poisoning in Western countries.^5,28,29,30^ Oil and gas combustion was the source of co poisoning for relatively few patients in the derivation cohort. The COGAS model tested in the oil and gas combustion-exposed subpopulation had high discrimination (eFigure 10 in the Supplement). We analyzed the verification power of COGAS score even if outcome was classified based on GDS score of 2 points. Furthermore, we created a prognostic prediction model based on two 2 GDS points similarly, and the results included all variables used to calculate COGAS score (eFigure 11 and eFigure 12 in the Supplement).

## Discussion

In this prognostic study, we first developed a new scoring model to stratify the risk of poor neurocognitive outcomes based on 5 variables at the early phase of acute co poisoning: older age (>50 years), low GCS score (≤12), shock, no HBO treatment, and creatine kinase (>320 U/L). The COGAS score for co poisoning was well validated externally with a separate cohort. So far, few studies have evaluated the risk factors for poor neurocognitive prognosis.^7,12,13,20,31^ To our knowledge, no study has created and validated an easily measurable scoring system with clinical and laboratory parameters for patients with co poisoning, regardless of severity and HBO therapy.

Previous studies had some limitations. In a multivariable logistic regression analysis conducted by Weaver et al,^12^ patients aged 36 years or older treated with HBO had reduced 6-week neurocognitive sequelae rates.^12^ Although the study was well conducted, the sample size of 75 patients was small; moreover, the study included patients who received HBO. Furthermore, Weaver et al^12^ did not suggest a scoring system for predicting poor prognosis. A study by Pan et al^13^ investigated 634 patients and found that GCS score, serum blood urea nitrogen level, and intubation days were associated with poor co poisoning prognosis. However, Pan et al^13^ included only patients who had received HBO treatment, and certain variables, such as the initial Hbco were not evaluated. The poor outcome was not well defined, as patients either still had sequelae, were bedridden, or had died, with no scoring model for prediction of poor outcomes. A study by Annane et al^31^ evaluated risk factors for outcomes after only mild co poisoning (those without loss of consciousness) using no scoring model. Studies by Choi^7^ and Min^20^ included relatively small sample sizes and only focused on estimating delayed neurocognitive sequelae. Moreover, the variables investigated were limited, with no scoring model. Others who studied the prognosis of co poisoning mainly analyzed risk factors for mortality and not neurocognitive prognosis.^32,33,34^ Furthermore, Nakajima et al^35^ assessed risk factors associated with in-hospital mortality, depressed mental status, and activities of daily living at discharge.

The 5 variables adopted in COGAS score were age older than 50 years, GCS score of 12 or less, presence of shock, no HBO treatment, and creatine kinase greater than 320 U/L. Old age is known to be a risk factor associated with poor prognosis.^7,12,20^ Decreased mental status has been investigated as a risk factor in previous studies,^13,15,16,18,19,36^ as in this study. However, any interval loss of consciousness was not a risk factor included in this study, which may mean that patients with prolonged rather than brief loss of consciousness were more likely to have a poor prognosis. Treatment with HBO was a remarkable factor, consistent with the findings reported in previous randomized clinical trials.^1,6,9,10^ In a few studies, serum creatine kinase^18,37,38,39,40^ was found to be a risk factor, as similarly observed in our study. We compared model 1 with model 2 (ie, with vs without laboratory values), and the score still had a high explanatory power of 0.812 (model 2), compared with 0.862 (model 1).

Previous studies^12,16,17^ have shown that longer co exposure intervals are associated with increased risk for neurocognitive sequelae. However, we did not use co exposure time to construct the main model because, for many patients, co exposure time was difficult to accurately measure in clinical settings. Addition of co exposure time in the scoring model was not suitable, although easier for the clinicians to use. We compared the diagnostic power of the model including co exposure time vs model 1 in the derivation cohort. There was no significant difference. In our analyses, there were several variables that were not found to be risk factors associated with 1-month cognitive sequelae after co exposure, including sex, intentionality, loss of consciousness, seizure, initial Hbco level, acidosis, lactate level, troponin I level, and history of cardiovascular or psychiatric disease. Early symptoms could not be investigated in patients who were unconscious. Furthermore, cognitive sequelae are not necessarily associated with physical expression at the time of poisoning.^7,12,20,41,42,43^ Although serum lactate and troponin I levels were reported as risk factors in previous studies,^19,44,45^ in this study, neither was associated with outcome. A few studies, such as a 2021 study by Sert et al,^17^ have suggested that acute brain damage detected with brain magnetic resonance imaging is associated with poor prognosis, but we excluded brain magnetic resonance imaging, as it cannot be performed early in all hospitals.

### Limitations

This study had some limitations. First, bias may have occurred because of excluded patients, despite the use of objective criteria. However, to our knowledge, this is the first study to create a screening model with a large sample size (>1000 patients). Second, this study only included patients in the Republic of Korea; therefore, COGAS score should be validated in different ethnic and regional groups. Third, charcoal was the most common co source in this study. However, in the oil and gas subpopulation, COGAS score had high verification power. Fourth, although a few studies conducted neurocognitive tests, usually equivalent to co batteries,^6,10^ we only evaluated outcomes with GDS scores. Many neurocognitive function tests may be difficult to perform in patients with sequelae. GDS score has the advantage of identifying neurocognitive functions, such as memory and concentration, as well as activities of daily living, and can be applied in all patients. We have previously reported GDS as a neurocognitive outcome in a study related to co poisoning,^25,46,47^ but it may be insufficient to assess neurological symptoms, such as motor weakness, speech disturbance, and peripheral neuropathy. However, a study by Choi et al^7^ reported neurologic impairment, including hemiplegia, speech disturbance, and peripheral neuropathy in 3 to 20 of 549 patients. We investigated neurological impairment, and the results did not change the outcome group of patients. Fifth, a new, potentially permanent mild cognitive decline or impairment may be considered a good outcome. However, our predictive model showed good performance even with GDS scores divided into 2.

## Conclusions

In this prognostic study a prediction model, dubbed COGAS score, using 5 factors associated with poor neurocognitive outcome at 1 month among patients with co poisoning was developed and externally validated. This scoring system showed excellent capacity to predict poor neurocognitive outcomes. Future studies are needed to validate COGAS score in various ethnic groups and other clinical applications.

## Notes

Supplement. **eAppendix 1.** Definition of the Study Variables **eAppendix 2.** Global Deterioration Scale Explanation **eTable.** Global Deterioration Scale Definitions **eFigure 1.** ROC Curve Analysis for Cutoff Value for Converting a Continuous Variable to a Categorical Variable **eFigure 2.** Flowcharts of the Derivation and Validation Cohorts **eFigure 3.** Histogram of the COGAS Score Model in the Derivation and Validation Cohorts **eFigure 4.** Calibration Plots of the COGAS Score for Each Cohort **eFigure 5.** Comparison of AUC Between Model 1 and Model 2 **eFigure 6.** Observed 1-Month Probability of Poor Neurocognitive Outcomes in the Derivation and Validation Cohorts According to the COGAS Score **eFigure 7.** Estimated 1-Month Probability of Poor Neurocognitive Outcomes by Model 2 and ROC Curve With the AUC of Model 2 **eFigure 8.** Calibration Plots of Model 2 in the Derivation and Validation Cohorts **eFigure 9.** Comparison Between the COGAS Score and the COGAS Score With the Presence of co Exposure Time **eFigure 10.** External Validation of the COGAS Score in the Oil and Gas Combustion-Exposed Subpopulation in the Derivation and Validation Cohorts **eFigure 11.** ROC Curve for the COGAS Scores as a Predictor When Neurocognitive Outcome Was Divided Based on 2 GDS Points **eFigure 12.** Estimated 1-Month Probability of Poor Neurocognitive Outcomes and ROC Curve With the AUC When Neurocognitive Outcome Was Divided Based on 2 GDS Points
eReferences
