# Supplementary material for: Derivation and Validation of a Score for Predicting Poor Neurocognitive Outcomes in Acute Carbon Monoxide Poisoning
Source: JAMA Netw Open. 2022 May 5;5(5):e2210552. doi: 10.1001/jamanetworkopen.2022.10552 (PMC9073567; doi:10.1001/jamanetworkopen.2022.10552)
Supplement: Supplement. — eAppendix 1. Definition of the Study Variables eAppendix 2. Global Deterioration Scale Explanation eTable. Global Deterioration Scale Definitions eFigure 1. ROC Curve Analysis for Cutoff Value for Converting a Continuous Variable to a Categorical Variable eFigure 2. Flowcharts of the Derivation and Validation Cohorts eFigure 3. Histogram of the COGAS Score Model in the Derivation and Validation Cohorts eFigure 4. Calibration Plots of the COGAS Score for Each Cohort eFigure 5. Comparison of AUC Between Model 1 and Model 2 eFigure 6. Observed 1-Month Probability of Poor Neurocognitive Outcomes in the Derivation and Validation Cohorts According to the COGAS Score eFigure 7. Estimated 1-Month Probability of Poor Neurocognitive Outcomes by Model 2 and ROC Curve With the AUC of Model 2 eFigure 8. Calibration Plots of Model 2 in the Derivation and Validation Cohorts eFigure 9. Comparison Between the COGAS Score and the COGAS Score With the Presence of co Exposure Time eFigure 10. External Validation of the COGAS Score in the Oil and Gas Combustion-Exposed Subpopulation in the Derivation and Validation Cohorts eFigure 11. ROC Curve for the COGAS Scores as a Predictor When Neurocognitive Outcome Was Divided Based on 2 GDS Points eFigure 12. Estimated 1-Month Probability of Poor Neurocognitive Outcomes and ROC Curve With the AUC When Neurocognitive Outcome Was Divided Based on 2 GDS Points eReferences [file jamanetwopen-e2210552-s001.pdf]

Supplemental Online Content

Kim SH, Lee Y, Kang S, Paik JH, Kim H, Cha YS. Derivation and validation of a score for predicting poor neurocognitive outcomes in acute carbon monoxide poisoning. *JAMA Netw Open*. 2022;5(5):e2210552. doi:10.1001/jamanetworkopen.2022.10552

eAppendix 1. Definition of the Study Variables

eAppendix 2. Global Deterioration Scale Explanation

eTable. Global Deterioration Scale Definitions

eFigure 1. ROC Curve Analysis for Cutoff Value for Converting a Continuous Variable to a Categorical Variable

eFigure 2. Flowcharts of the Derivation and Validation Cohorts

eFigure 3. Histogram of the COGAS Score Model in the Derivation and Validation Cohorts

eFigure 4. Calibration Plots of the COGAS Score for Each Cohort

eFigure 5. Comparison of AUC Between Model 1 and Model 2

eFigure 6. Observed 1-Month Probability of Poor Neurocognitive Outcomes in the Derivation and Validation Cohorts According to the COGAS Score

eFigure 7. Estimated 1-Month Probability of Poor Neurocognitive Outcomes by Model 2 and ROC Curve With the AUC of Model 2

eFigure 8. Calibration Plots of Model 2 in the Derivation and Validation Cohorts

eFigure 9. Comparison Between the COGAS Score and the COGAS Score With the Presence of CO Exposure Time

eFigure 10. External Validation of the COGAS Score in the Oil and Gas Combustion-Exposed Subpopulation in the Derivation and Validation Cohorts

eFigure 11. ROC Curve for the COGAS Scores as a Predictor When Neurocognitive Outcome Was Divided Based on 2 GDS Points

eFigure 12. Estimated 1-Month Probability of Poor Neurocognitive Outcomes and ROC Curve With the AUC When Neurocognitive Outcome Was Divided Based on 2 GDS Points

eReferences

This supplemental material has been provided by the authors to give readers additional information about their work.

© 2022 Kim SH et al. *JAMA Network Open.*

eAppendix 1. Definition of the Study Variables

Carbon monoxide (CO) exposure duration, as reported by the patient or patients' guardians, was the expected maximum duration of CO exposure, measured from the time of normal state of consciousness to patient rescue. Any state of loss of consciousness was defined as a case of such, regardless of the length of loss of consciousness. Shock was diagnosed when a vasopressor was required to resuscitate the patient and if lactate levels exceeded 2.0 mmol/L.

© 2022 Kim SH et al. *JAMA Network Open.*

eAppendix 2. Global Deterioration Scale Explanation

The Global Deterioration Scale (GDS) is a validated, reliable instrument for describing the clinical progression of dementia.[1] It is also used to determine the prognosis of patients with carbon monoxide (CO) poisoning,[2-4] severe chronic obstructive pulmonary disease, Alzheimer's disease, and vasculopathy-related dementia.[1,5-7]

Although the GDS score is not as diverse as a CO battery, it has the advantage of being able to identify neurocognitive functions, such as memory and concentration, as well as activities of daily living, through interviews. Moreover, many neurocognitive function tests may be difficult for patients with sequelae. The Short-Form General Health Survey-36, a commonly used testing tool, has a set of self-reported questions; however, it is limited in evaluating patients with severe neurological impairment as it requires an individual's ability to understand and address the questions. Digit span, trail making, and clock drawing are good evaluation tools but require short-term memory and visuospatial functions. Therefore, the GDS score can be used for all patients with CO poisoning regardless of poisoning severity. The scale consists of seven stages, with higher scores indicating greater severity.

© 2022 Kim SH et al. *JAMA Network Open.*

eTable. Global Deterioration Scale Definitions

Stage	Cognitive dysfunction	Clinical characteristics
1	No cognitive decline	<u>*Patients appear clinically normal.*</u><br><br>No complaints of memory deficits.<br><br>No evident memory deficit on clinical interview.
2	Very mild cognitive decline	<u>*Patients complain of memory deficits.*</u><br><br>Most frequently, patients:<br><br>(a) forget where they have placed familiar objects<br><br>(b) forget the name of someone they formerly knew well.<br><br>No objective evidence of memory deficit on clinical interview.<br><br>No objective deficits in employment or social situations.<br><br>Patients display appropriate concern about their symptoms.
3	Mild cognitive decline	<u>*Earliest clear-cut deficits*</u>.<br><br>Objective evidence of memory deficit was obtained only through an intensive interview conducted by a trained geriatric psychiatrist. Concentration deficit may be evident on clinical testing.<br><br>Patients may demonstrate a reduced ability to:<br><br>(a) remember names upon introduction to new people<br><br>(b) retain information after reading a passage from a book<br><br>Decreased performance may be manifested in demanding employment and social situations. Examples may include the following:<br><br>(a) coworkers becoming aware of the patient's relatively poor performance<br><br>(b) difficulties in finding words and names becoming evident to intimate acquaintances<br><br>(c) losing or misplacing objects of value<br><br>(d) getting lost when traveling to unfamiliar locations<br><br>The subtlety of the clinical symptoms may be exacerbated by denial that is often manifested by these patients. Mild-to-moderate anxiety also accompanies the symptoms, typically when the patients are forced to cope with challenging employment and social demands that render them unable to negotiate.

© 2022 Kim SH et al. *JAMA Network Open.*

Stage	Cognitive dysfunction	Clinical characteristics
4	Moderate cognitive decline	<u>*Clear-cut deficits on careful clinical interview.*</u><br><br>Deficits may be manifested in several areas:<br><br>(a) concentration deficit elicited in serial subtractions<br><br>(b) decreased knowledge of current and recent life events<br><br>(c) upon careful questioning, patients may exhibit difficulty recalling their personal history<br><br>(d) decreased ability to travel alone and manage finances<br><br>Patients can no longer perform complex tasks accurately and efficiently; however, certain abilities remain preserved:<br><br>(a) orientation to time and people<br><br>(b) familiar persons and faces can be distinguished from strangers<br><br>(c) ability to travel to familiar locations<br><br>Denial is often the dominant defense mechanism. The evident decline in patients' intellectual and cognitive capacities is too overwhelming with a loss of full conscious acceptance and recognition. Flattening of effect and withdrawal from previously challenging situations is observed.
5	Moderately severe cognitive decline	<u>*Patients can no longer survive without some assistance.*</u><br><br>During interviews, patients are unable to recall a major relevant aspect of their current lives. Examples include the following:<br><br>(a) difficulty recalling their address or telephone number, names of close family members, such as grandchildren, or the name of the high school or university from which they graduated<br><br>(b) some disorientation with time (date, day of the week, season) or location<br><br>(c) well-educated patients may have difficulty counting backwards from 40 by fours or from 20 by twos.<br><br>Patients retain the knowledge of many major facts regarding themselves and others. They invariably know their own names and generally know their spouse and children's names. They require no assistance with toileting and eating but may have some difficulty choosing the proper clothing to wear and may occasionally clothe themselves improperly (e.g., put their shoes on the wrong feet).

© 2022 Kim SH et al. *JAMA Network Open.*

Stage	Cognitive dysfunction	Clinical characteristics
6	Severe cognitive decline	*Patients may occasionally forget the name of their spouse, on whom they depend entirely for survival.*<br><br>Patients are largely unaware of all recent events and experiences in their lives.<br><br>They retain some knowledge of their past but is very uncertain. They are generally unaware of their surroundings, the year, or the season and may have difficulty counting backward, and sometimes forward, from 10. Patients require substantial assistance with activities of daily living. These symptoms are quite variable and include the following:<br><br>(a) delusional behavior (e.g., patients may accuse their spouse of being an impostor, may talk to imaginary figures in the environment, or their own reflection in the mirror)<br><br>(b) obsessive symptoms (e.g., continual repetition of simple cleaning activities)<br><br>(c) anxiety symptoms, agitation, and previously nonexistent violent behavior<br><br>(d) cognitive abulia (i.e., loss of willpower because they cannot dwell on a thought long enough to determine a purposeful course of action).
7	Very severe cognitive decline	*All verbal abilities are lost.*<br><br>Frequently, there is no speech ability at all; only grunting remains.<br><br>Patients have urinary incontinence and require assistance with toileting and eating. They lose psychomotor skills (e.g., the ability to walk). The brain may find it difficult to tell the body what to do. Generalized cortical and focal neurologic signs and symptoms are frequently present.

© 2022 Kim SH et al. *JAMA Network Open.*

eFigure 1. ROC Curve Analysis for Cutoff Value for Converting a Continuous Variable to a Categorical Variable

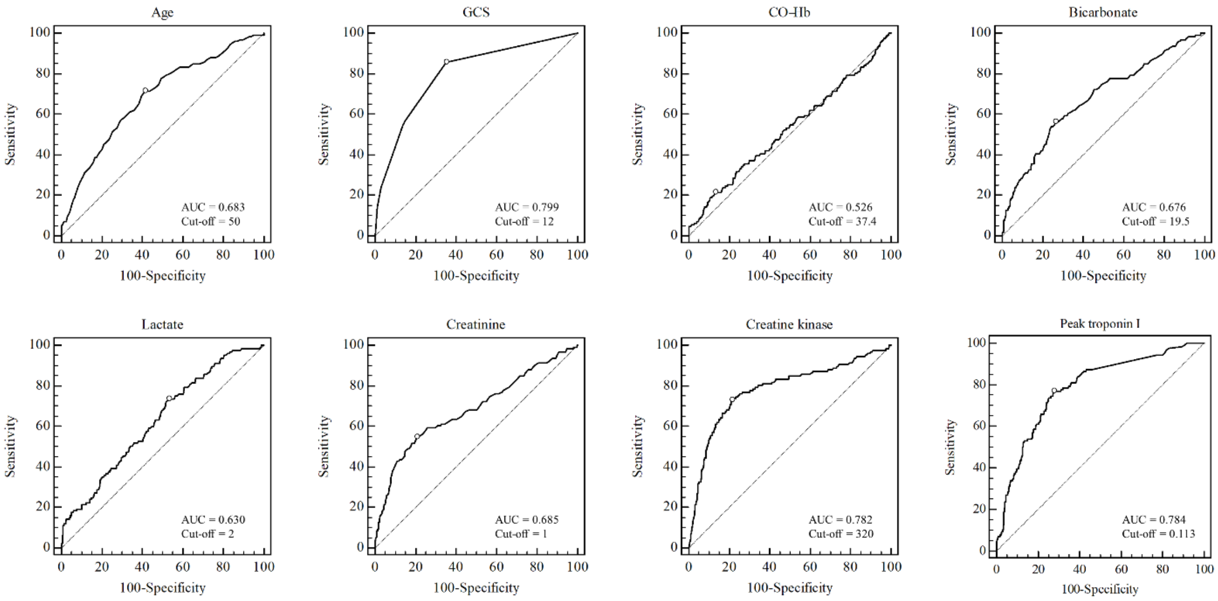

GCS = Global Deterioration Scale; CO-Hb = carboxyhemoglobin; ROC = receiver operating characteristic; AUC = area under the ROC curve.

© 2022 Kim SH et al. *JAMA Network Open.*

eFigure 2. Flowcharts of the Derivation and Validation Cohorts

(A)

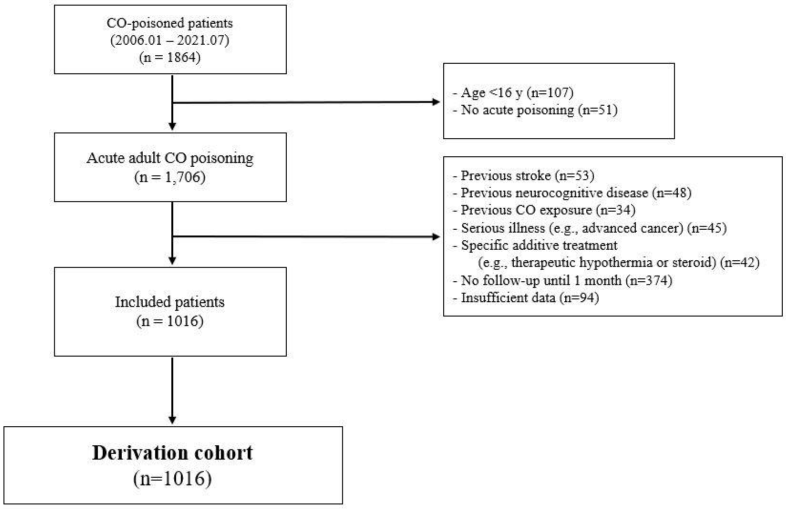

(B)

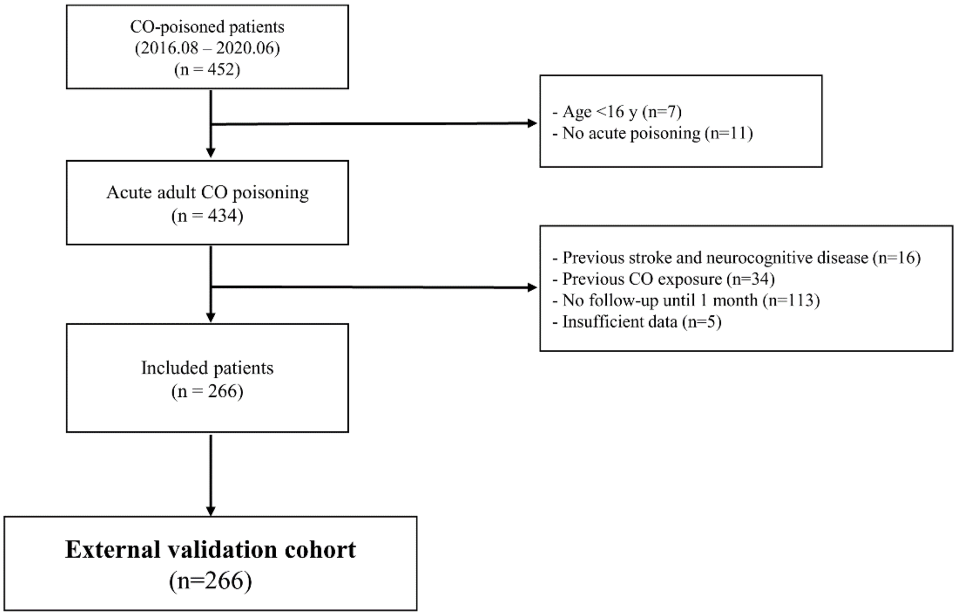

CO = carbon monoxide.

© 2022 Kim SH et al. *JAMA Network Open.*

eFigure 3. Histogram of the COGAS Score Model in the Derivation and Validation Cohorts

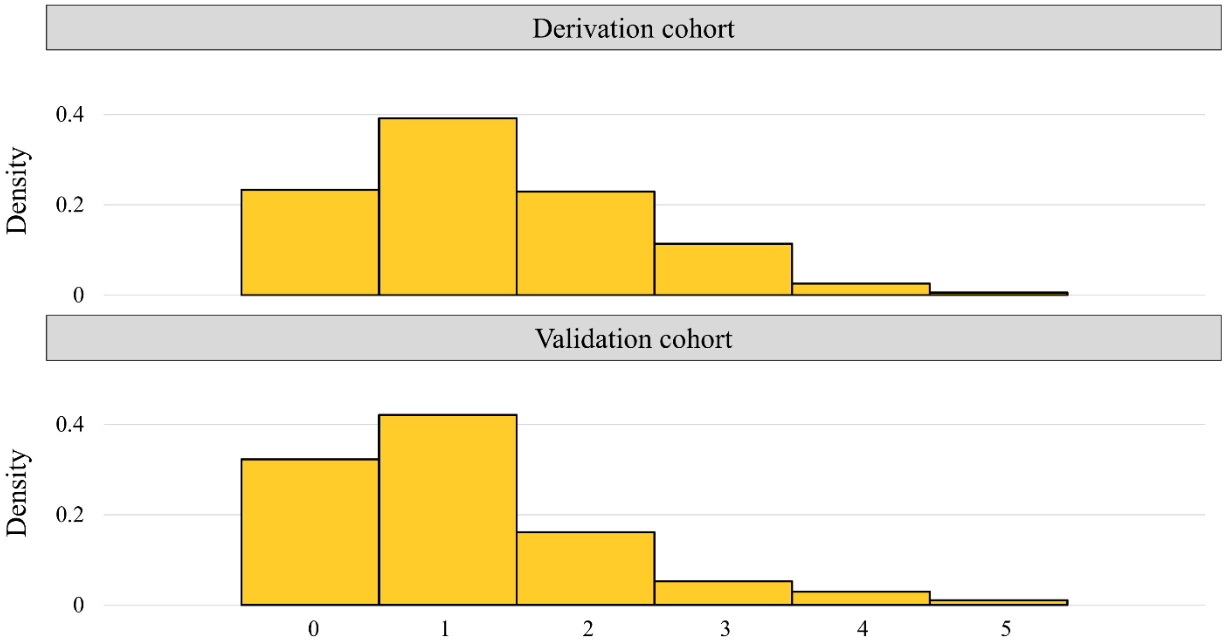

COGAS = Creatine kinase, hyperbaric Oxygen therapy, Glasgow coma scale, Age, Shock.

© 2022 Kim SH et al. *JAMA Network Open.*

eFigure 4. Calibration Plots of the COGAS Score for Each Cohort

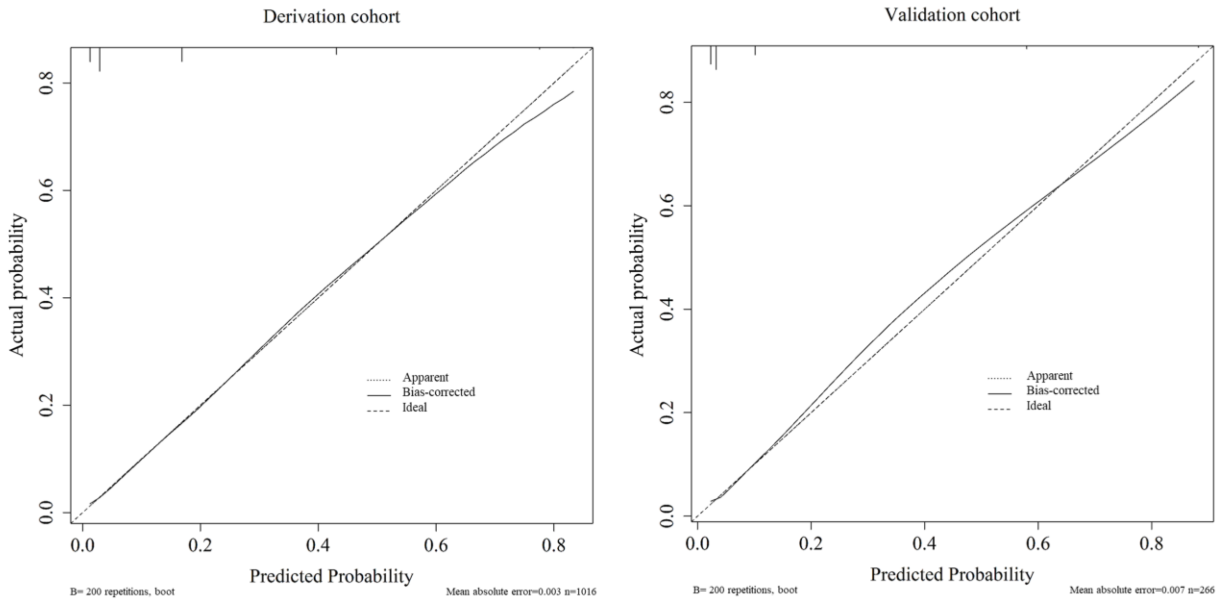

COGAS = Creatine kinase, hyperbaric Oxygen therapy, Glasgow coma scale, Age, Shock.

© 2022 Kim SH et al. *JAMA Network Open.*

eFigure 5. Comparison of AUC Between Model 1 and Model 2

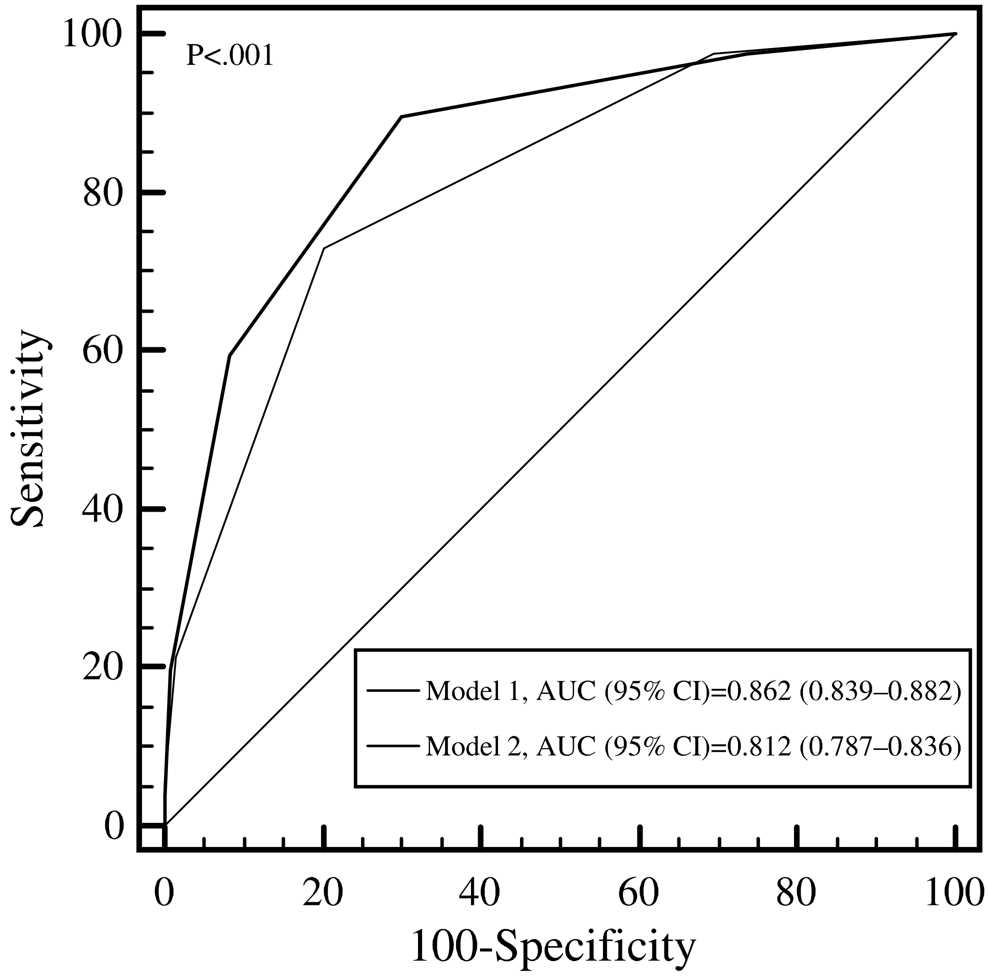

AUC = area under the ROC curve

© 2022 Kim SH et al. *JAMA Network Open.*

eFigure 6. Observed 1-Month Probability of Poor Neurocognitive Outcomes in the Derivation and Validation Cohorts According to the COGAS Score

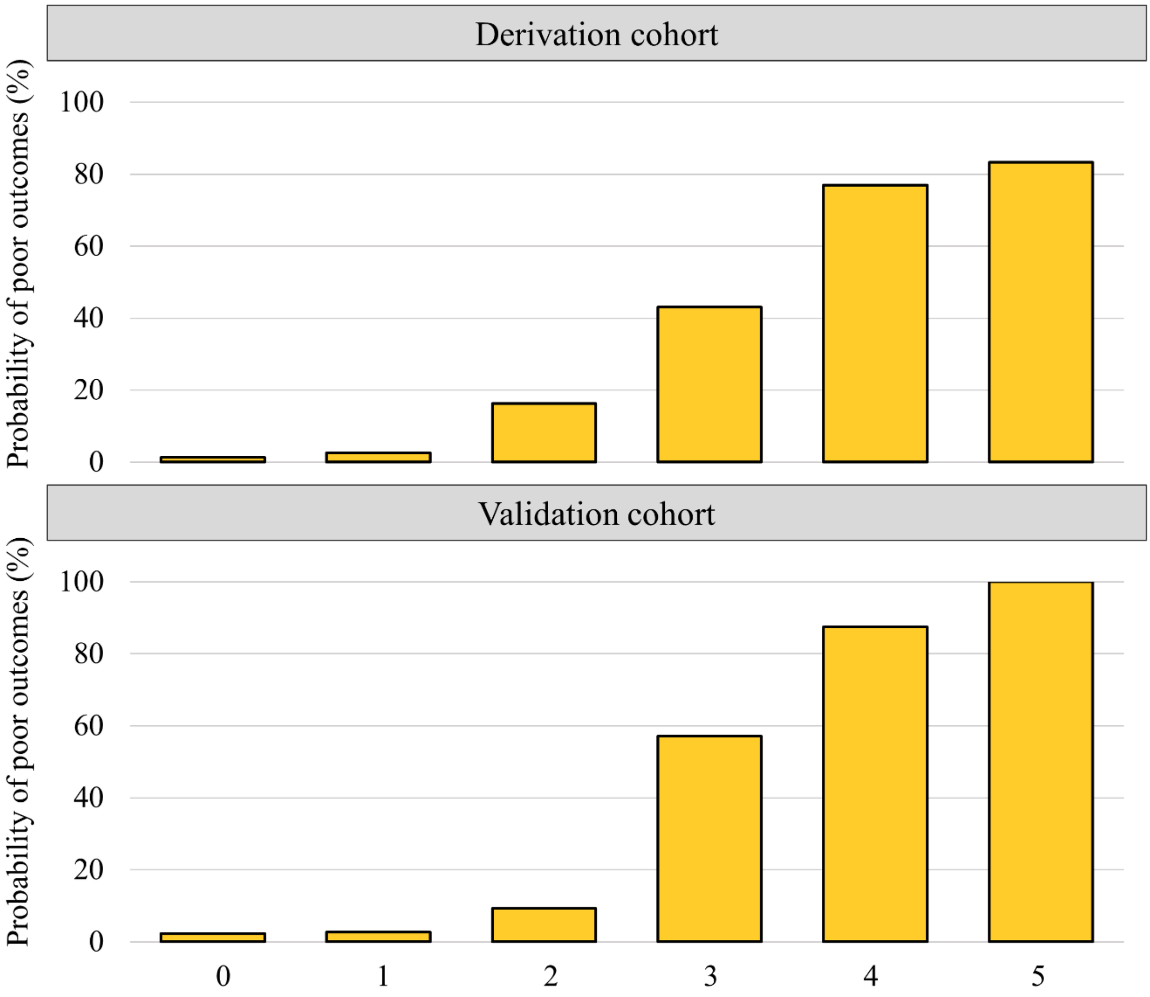

COGAS = Creatine kinase, hyperbaric Oxygen therapy, Glasgow coma scale, Age, Shock.

© 2022 Kim SH et al. *JAMA Network Open.*

eFigure 7. Estimated 1-Month Probability of Poor Neurocognitive Outcomes by Model 2 and ROC Curve With the AUC of Model 2

(A)

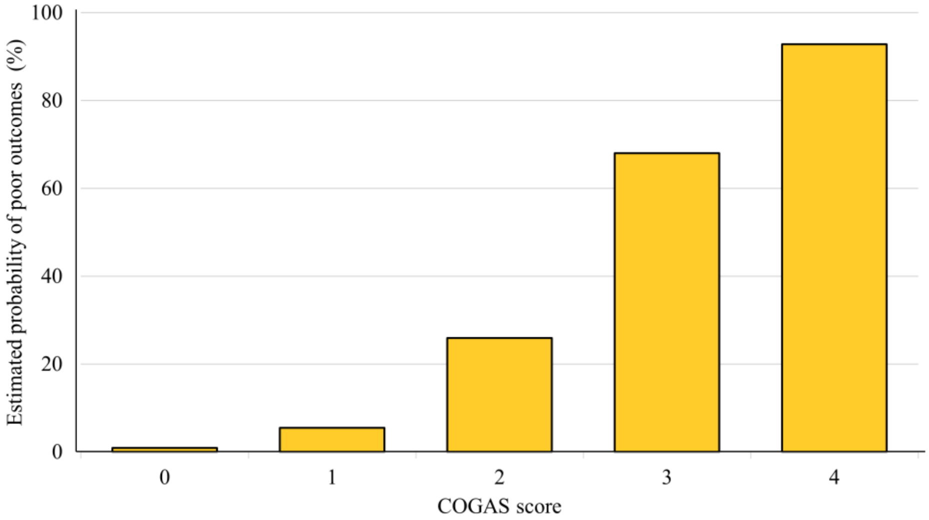

(B)

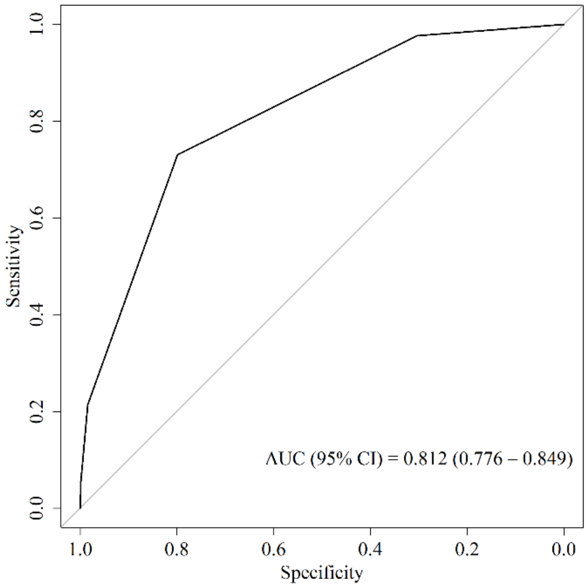

In the score developed by the variables of model 2, the observed 1-month probabilities of poor neurocognitive outcomes were similar in both cohorts, and the estimated 1-month probabilities of poor neurocognitive outcomes were as follows: 0%, 5.5%, 25.9%, 68.0%, 92.8% for a score of 1,2,3 and 4, respectively. The observed and estimated probabilities of poor neurocognitive outcomes were strongly correlated; correlation coefficient in model 1, r=0.989, R2=0.978, *P*<.001; in model 2, r=0.969, R2=0.939, *P*<.001.

COGAS = Creatine kinase, hyperbaric Oxygen therapy, Glasgow coma scale, Age, Shock; ROC = receiver operating characteristic; AUC = area under the ROC curve.

© 2022 Kim SH et al. *JAMA Network Open.*

eFigure 8. Calibration Plots of Model 2 in the Derivation and Validation Cohorts

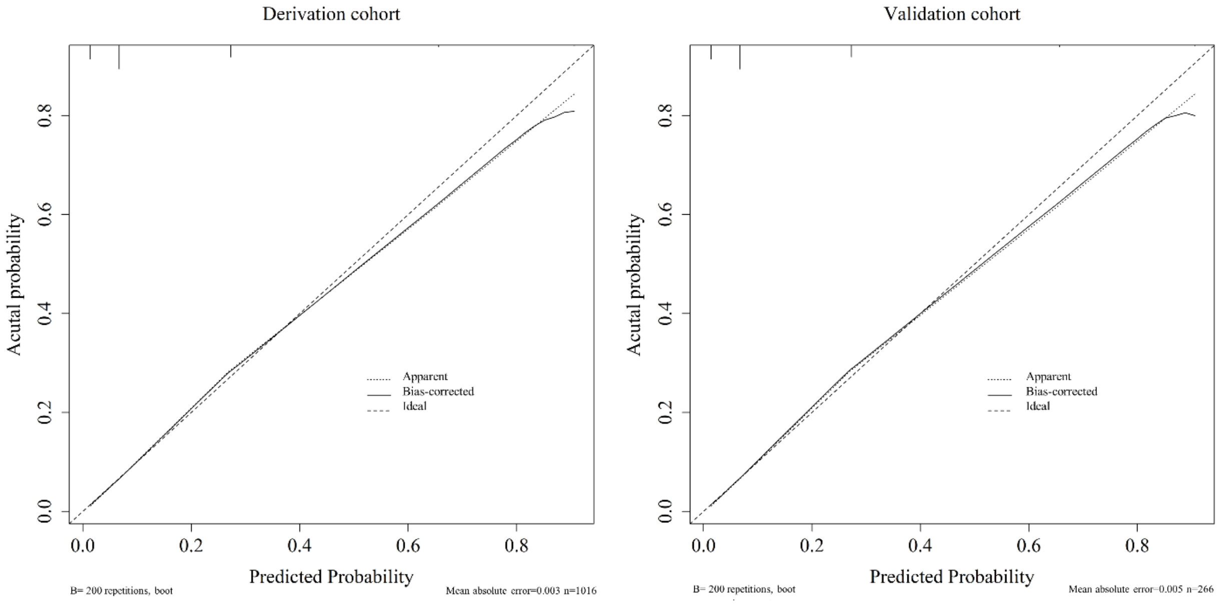

© 2022 Kim SH et al. *JAMA Network Open.*

eFigure 9. Comparison Between the COGAS Score and the COGAS Score With the Presence of CO Exposure Time

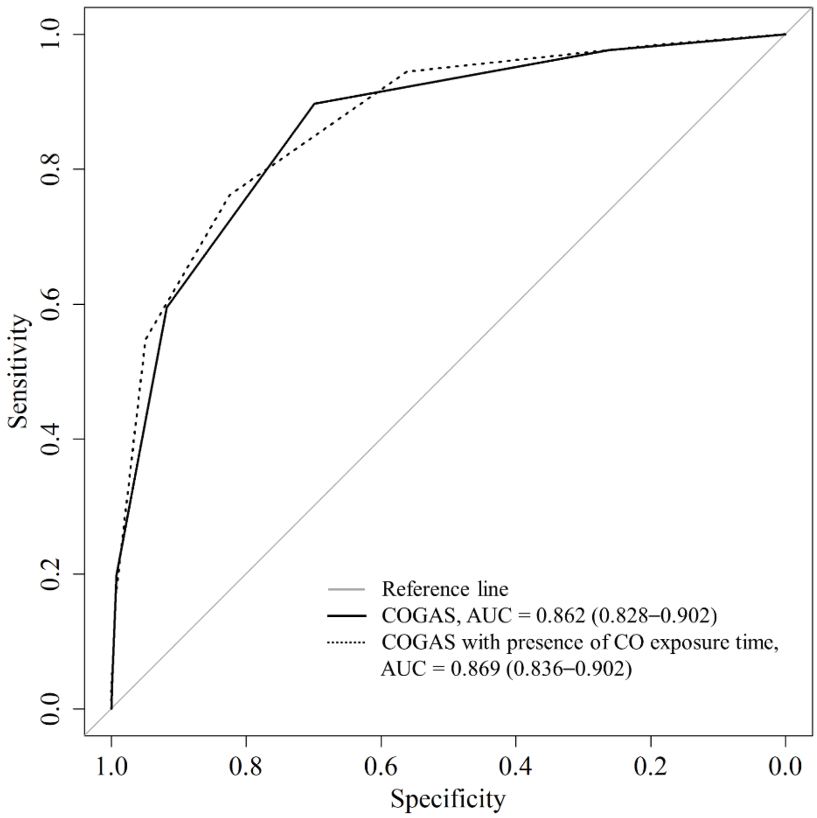

CO = carbon monoxide; COGAS = Creatine kinase, hyperbaric Oxygen therapy, Glasgow coma scale, Age, Shock; AUC = area under the ROC curve.

© 2022 Kim SH et al. *JAMA Network Open.*

eFigure 10. External Validation of the COGAS Score in the Oil and Gas Combustion-Exposed Subpopulation in the Derivation and Validation Cohorts

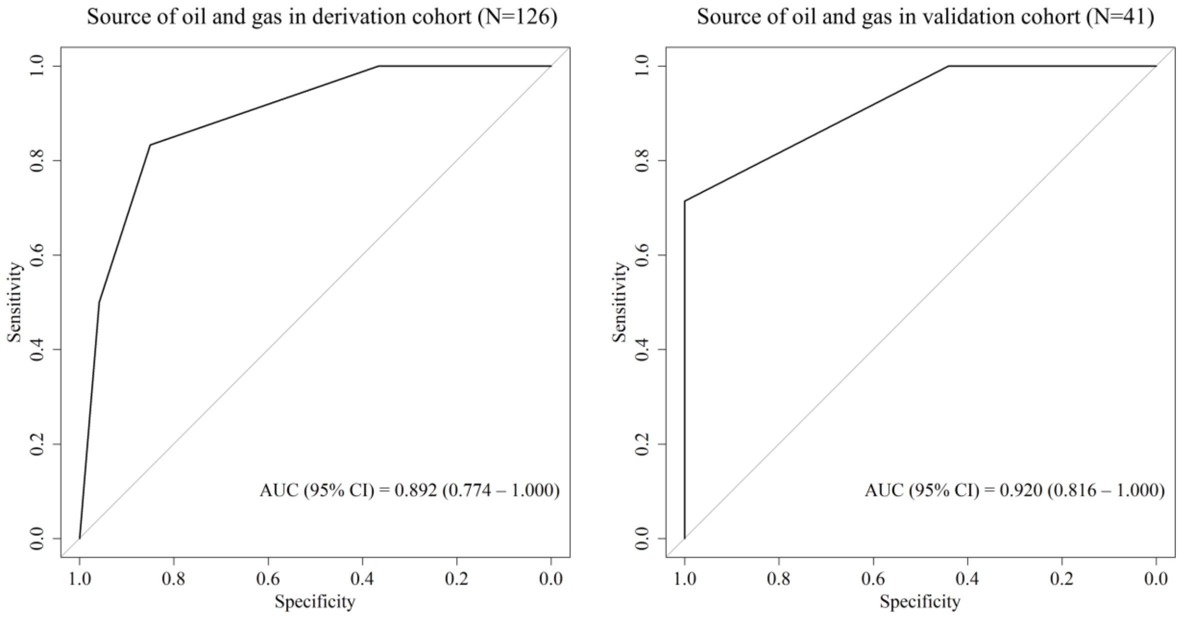

COGAS = Creatine kinase, hyperbaric Oxygen therapy, Glasgow coma scale, Age, Shock; AUC = area under the ROC curve; CI = confidence interval

© 2022 Kim SH et al. *JAMA Network Open.*

eFigure 11. ROC Curve for the COGAS Scores as a Predictor When Neurocognitive Outcome Was Divided Based on 2 GDS Points

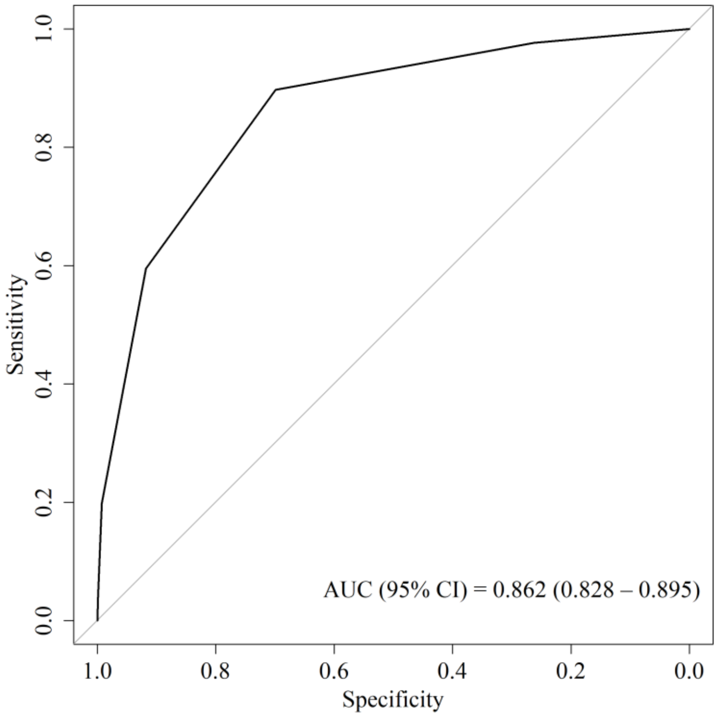

ROC = receiver operating characteristic; COGAS = Creatine kinase, hyperbaric Oxygen therapy, Glasgow coma scale, Age, Shock; AUC = area under the ROC curve; CI = confidence interval

© 2022 Kim SH et al. *JAMA Network Open.*

eFigure 12. Estimated 1-Month Probability of Poor Neurocognitive Outcomes and ROC Curve With the AUC When Neurocognitive Outcome Was Divided Based on 2 GDS Points

(A)

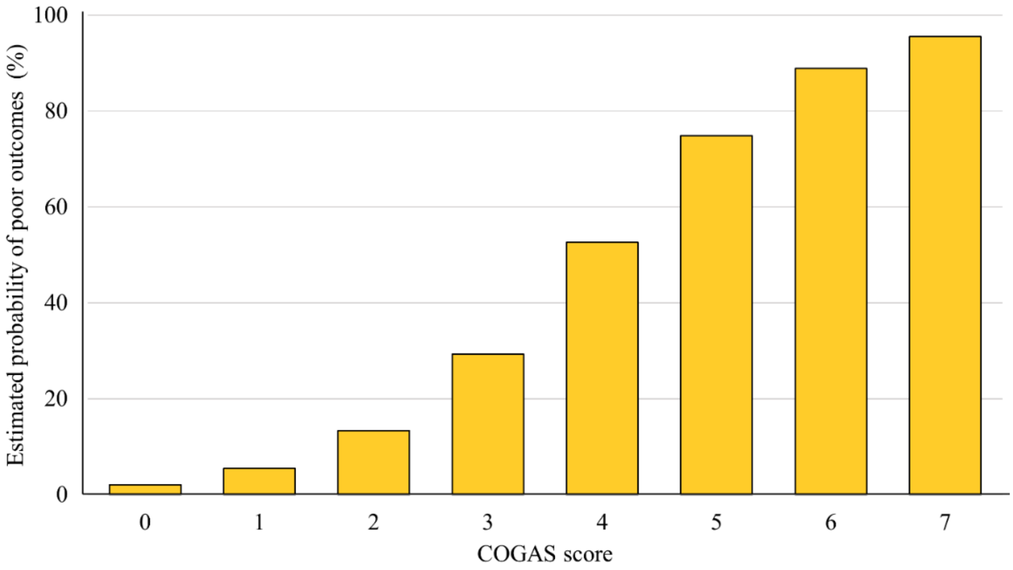

(B)

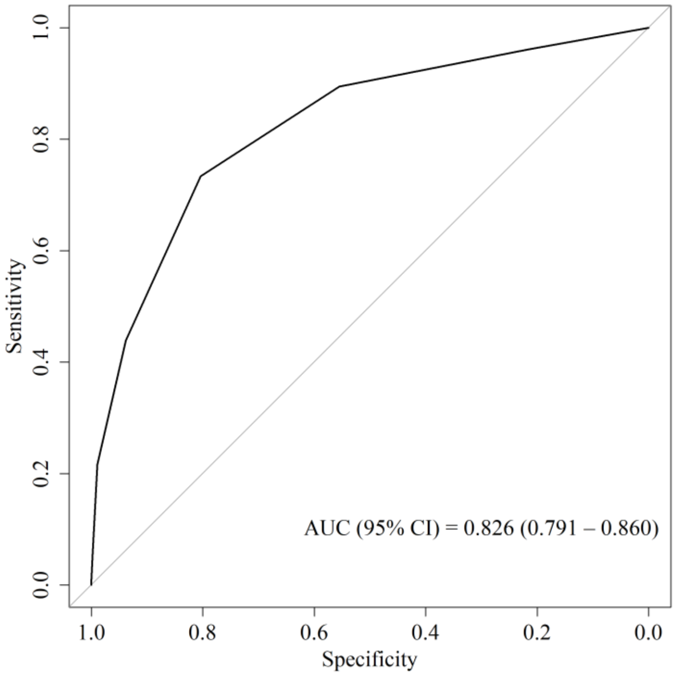

Variables included in this model were as follows: older age (>50 years), low GCS (≤12), shock, no use of hyperbaric oxygen therapy, creatine kinase (>320 U/L), hypertension, and serum bicarbonate (≤19.6 mmol/L).

ROC = receiver operating characteristic; AUC = area under the ROC curve; GDS = Global Deterioration Scale

© 2022 Kim SH et al. *JAMA Network Open.*

eReferences

1. Reisberg B, Ferris SH, de Leon MJ, Crook T. The Global Deterioration Scale for assessment of primary degenerative dementia. *Am J Psychiatry.* 1982;139(9):1136-1139. doi:10.1176/ajp.139.9.1136
2. Kim SJ, Thom SR, Kim H, et al. Effects of adjunctive therapeutic hypothermia combined with hyperbaric oxygen therapy in acute severe carbon monoxide poisoning. *Crit Care Med.* 2020;48(8):e706-e714. doi:10.1097/ccm.0000000000004419
3. Cho DH, Ko SM, Son JW, Park EJ, Cha YS. Myocardial injury and fibrosis from acute carbon monoxide poisoning: a prospective observational study. *JACC Cardiovasc Imaging.* 2021;14(9):1758-1770. doi:10.1016/j.jcmg.2021.02.020
4. Lee Y, Cha YS, Kim SH, Kim H. Effect of Hyperbaric Oxygen Therapy Initiation Time in Acute Carbon Monoxide Poisoning. *Crit Care Med.* 2021;49(10):e910-e919. doi:10.1097/CCM.0000000000005112
5. Paul RH, Cohen RA, Moser DJ, et al. The global deterioration scale: relationships to neuropsychological performance and activities of daily living in patients with vascular dementia. *J Geriatr Psychiatry Neurol.* 2002;15(1):50-54. doi:10.1177/089198870201500110
6. Eisdorfer C, Cohen D, Paveza GJ, et al. An empirical evaluation of the Global Deterioration Scale for staging Alzheimer's disease. *Am J Psychiatry.* 1992;149(2):190-194. doi:10.1176/ajp.149.2.190
7. Ozge C, Ozge A, Unal O. Cognitive and functional deterioration in patients with severe COPD. *Behav Neurol.* 2006;17(2):121-130. doi:10.1155/2006/848607

© 2022 Kim SH et al. *JAMA Network Open.*
